# *Toxoplasma gondii* infection in wild mustelids and cats across an urban-rural gradient

**DOI:** 10.1371/journal.pone.0199085

**Published:** 2018-06-20

**Authors:** Macarena Barros, Oscar Cabezón, Jitender P. Dubey, Sonia Almería, María P. Ribas, Luis E. Escobar, Barbara Ramos, Gonzalo Medina-Vogel

**Affiliations:** 1 Centro de Investigacion para la Sustentabilidad, Universidad Andres Bello, República, Santiago, Chile; 2 UAB, Centre de Recerca en Sanitat Animal (CReSA, IRTA-UAB), Campus de la Universitat Autònoma de Barcelona, Bellaterra, Spain; 3 Servei d'Ecopatologia de Fauna Salvatge, Departament de Medicina I Cirugia Animals, Universitat Autònoma de Barcelona, Bellaterra, Spain; 4 Animal Parasitic Diseases Laboratory, Beltsville Agricultural Research Center, Agricultural Research Service, United States Department of Agriculture, Beltsville, Maryland, United States of America; 5 Departament de Sanitat I d´Anatomia Animals, Universitat Autònoma de Barcelona, Bellaterra, Spain; 6 Department of Fish and Wildlife Conservation, Virginia Tech., Blacksburg, Virginia, United States of America; NIH, UNITED STATES

## Abstract

The increase in human population and domestic pets, such as cats, are generating important consequences in terms of habitat loss and pathogen pollution of coastal ecosystems with potential to generate negative impacts in marine biodiversity. *Toxoplasma gondii* is the etiological agent of zoonotic disease toxoplasmosis, and is associated with cat abundance and anthropogenic disturbance. The presence of *T*. *gondii* oocysts in the ocean has negatively affected the health status of the threatened Southern sea otter (*Enhydra lutris nereis*) populations. The present study analyzed seroprevalence and presence of *T*. *gondii* DNA in American mink (*Neovison vison)*, Southern river otters (*Lontra provocax)* and domestic cats (*Felis silvestris catus*) in four different areas in Southern Chile comprising studies in rivers and lakes in Andean foothills and mountains, marine habitat and island coastal ecosystems. Mean seroprevalence of *T*. *gondii* in the study was 64% of 151 total animals sampled: 59% of 73 American mink, 77% of 13 Southern river otters, 68% of 65 domestic cats and in two of two kodkods (*Leopardus guigna*). *Toxoplasma gondii* DNA was detected in tissues from one American mink and one Southern river otter. The present study confirms the widespread distribution of *T*. *gondii* in Southern Chile, and shows a high exposure of semiaquatic mustelids and domestic cats to the parasite. Cats and anthropogenic disturbance have a role in the maintenance of *T*. *gondii* infection in ecosystems of southern Chile.

## Introduction

Contamination of the aquatic environment derived from human activities is a concern worldwide, and the study of the biological effects of these activities in the marine ecosystem has been declared a goal for future research by the scientific community [1]. The effect of human settlement in coastal areas causes significant negative impacts on coastal marine habitats and their wildlife species, including the flow of human/terrestrial pathogens [2, 3, 4]. *Toxoplasma gondii*, a worldwide distributed zoonotic protozoan parasite, presents an indirect cycle with domestic and wild felines as definitive hosts. Oocysts are eliminated to the environment through feces where they can remain infective for months or years. Warm-blooded species can become infected by ingesting the sporulated oocysts and infection can persist in infected hosts for the life of the host [5]. Although better studied in terrestrial landscapes, *T*. *gondii* has also emerged as a significant aquatic pathogen linked to marine mammal infection and water-borne outbreaks of disease in humans worldwide [6]. The presence of *T*. *gondii* in the marine and freshwater ecosystems has been confirmed by the exposure of several aquatic species to this parasite [7, 8, 9, 10], with clinical disease observed in seals, dolphins, whales, sea otters, and manatees [7, 11, 12]. Animals living in freshwater systems are also at risk. As many as 85% (82 of 95) of free-ranging Amazon river dolphins had antibodies to *T*. *gondii* [13]. In this respect, *T*. *gondii* has been used as a model for the study of land-to-sea pathogen contamination [14, 15, 16]. Higher flow of *T*. *gondii* oocysts to the marine ecosystem has been related to the high coastal human densities and estuarine wetland degradation due to human activities [14, 17]. The effect of this higher *T*. *gondii* presence in the ocean has negatively affected the health status of the threatened Southern sea otter (*Enhydra lutris nereis*) populations, increasing its mortality related to the parasite [18, 19], and supporting the hypothesis of land to sea transmission of this parasite through contaminated freshwater runoff [20]. Widespread *T*. *gondii* infection in aquatic mammals suggests that contamination of terrestrial watersheds with *T*. *gondii* is prevalent, and that sufficient numbers of oocysts are distributed in freshwater and marine ecosystems to infect and cause disease in both near-shore and pelagic mammals [10].

Chile has several aquatic wild carnivore endemic species classified as endangered by the International Union for the Conservation of Nature (IUCN) [21]. One of the most important species is the Southern river otter (*Lontra provocax*, referred here as otter), a semi-aquatic species that is a top predator of river and marine fauna [22] and is considered to be the otter with the smallest distribution in the world [23, 24]. The distribution of this otter has declined drastically due to combined pressures from the destruction of habitat, removal of vegetation, river and stream canalization, and extensive dredging [24, 25]. This species is considered “endangered” by the IUCN Red List of Threatened Species [21]. This otter shares its ecosystem with the American mink (*Neovison vison*), a widely distributed exotic invasive mustelid present worldwide [26, 27]. American mink can negatively impact otter populations in different ways, including competition for resources and disease transmission. In this way, the presence of other species, such as the mink, can serve as reservoirs of diseases for the otters. For example, the exposure of the otter to *T*. *gondii* will be influenced by the presence of felids in the field. In this respect, the domestic and feral cats represent the most important epidemiological factor in the dispersion of the parasite [28] and its distribution and density is directly influenced by human presence. In Southern Chile, there may be other potential felid definitive *T*. *gondii* hosts such as the kodkod (*Leopardus guigna*). The role of this species in the epidemiology of *T*. *gondii* has not been evaluated. Kodkod is the smallest wild felid in the Americas. It lives primarily in central and southern Chile and marginally in adjoining areas of Argentina. Its area of distribution is small compared to the other South American felids. Since 2002, it has been listed as Vulnerable on the IUCN Red List as the total effective population may comprise less than 10,000 mature individuals, and is threatened due to persecution and loss of habitat and prey base [21].

The epidemiology of *T*. *gondii* is understudied in Chile. Although some studies have reported its circulation in human beings, domestic animals, and wildlife, these studies have been undertaken in few species and geographic areas [29, 30–34]. Therefore, the objective of the present study was to analyze *T*. *gondii* distribution in the diverse terrestrial/aquatic interfaces in several areas of Southern Chile, particularly in relation to their proximity to human settlements and the presence of domestic cats, assaying DNA and antibodies against this parasite in semi-aquatic carnivores.

## Materials and methods

### Study areas

**Fig 1:**
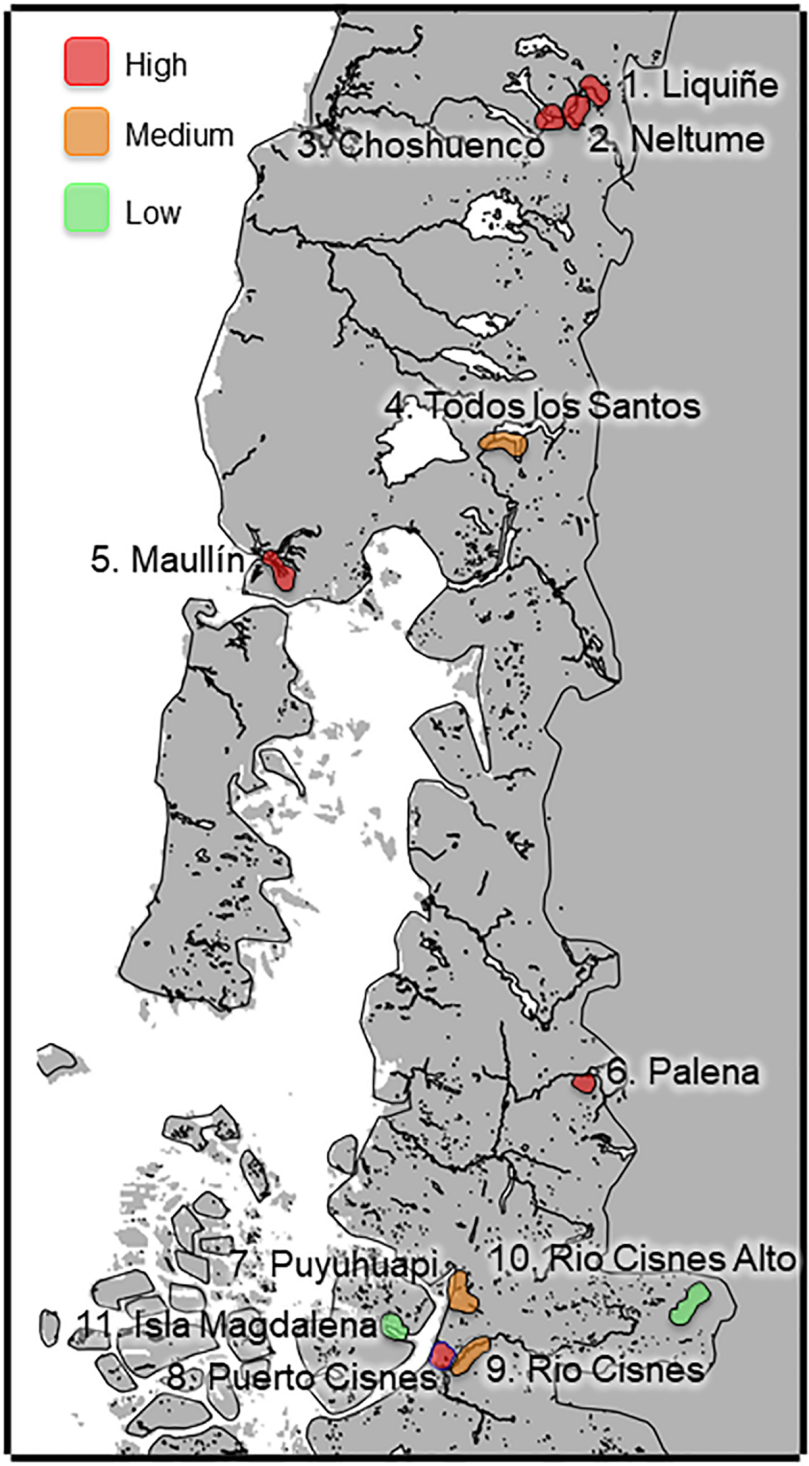
Study area. Eleven study sites, grouped into four study areas in Southern Chile, between latitude 39°-45° S. Area 1, represents an Andean foothill ecosystem (1:Liquiñe, 2:Neltume, 3:Choshuenco, 4:Todos los Santos); area 2, represents a marine coastal ecosystem (5:Maullín, 7:Puyuhuapi, 8:Puerto Cisnes, 9:Rio-Cisnes); area 3, represents Southern Andean mountain valleys (6:Palena, 10:Rio-Cisnes-Alto); area 4, represents an island (11:Magdalena Island). Red: High degree of presence human-domestic cat. Orange: Medium degree of presence human-domestic cat. Green: Low degree of presence human-domestic cat.

Eleven study sites were chosen according to human, cat, mink and otter presence and then grouped into four study areas according to landscape characteristics in Southern Chile between 39°-45° S latitudes (Fig 1).

**Area 1** (39.3° - 41.3°S; 200–300 m.a.s.l; sites 1 to 4; Fig 1) represents an Andean foothill ecosystem and comprised the Liquiñe river, Neltume lake, Choshuenco lake and Todos-los-Santos lake. This area has a cold temperate rainy Mediterranean climate. The annual average temperature is low (11°C) and falls toward the south, with strong daily thermal oscillation and high rainfall throughout the year. In winter, the precipitation increases and there are almost no dry months. The vegetation corresponds to Andean rainforest of deciduous and coniferous trees, comprising *Nothofagus dombeyi*, *Podocarpus nubigenus*, *Aextoxicon punctatum*, and *Drymis winteri* [35]. The four sites sampled had little slope. The landscape has residential, tourist and agricultural use with pervious surfaces.

**Area 2** (41.5° - 44.8°S; 20–80 m.a.s.l; sites 5, 7, 8 and 9; Fig 1) represents a marine coastal ecosystem and comprises the Puerto-Cisnes and Puyuhuapi fiords, the Maullín, and Rio-Cisnes rivers. This area has a rainy temperate maritime climate without a dry season. The area is characterized by low temperatures, with an average annual temperature of 10.2°C. Rainfall is abundant and present throughout the year. The vegetation present in Maullín is the same as Area 1, while in Puerto-Cisnes is the Patagonian steppe [35]. The Maullín, Puyuhuapi and Rio-Cisnes areas all have residential, tourist, and agricultural use with pervious surfaces. Puerto-Cisnes is a residential area with primarily impervious surface.

**Area 3** (43.3° - 44.6°S; 250–750 m.a.s.l; sites 6 and 10; Fig 1) represents Southern Andean mountain valleys and is comprised of the Palena and Rio-Cisnes-Alto rivers. The Palena river has a microclimate with average annual temperatures of 9.5°C, with average rainfall of 1700mm. The Rio-Cisnes-Alto river has a cold steppe climate affected by the eastern slope of the Andes. The average annual temperature is 7.1°C. Rainfall decreases during autumn, and in winter snow precipitation occurs. The vegetation type is tundra [35]. This area has little slope. The landscape has residential, tourist and agricultural use with pervious surfaces.

**Area 4** (44.2° - 44.5°S; 10 m.a.s.l; site 11; Fig 1): represents an island. Magdalena Island has rainy conditions, with low average annual temperature, ranging between 4°C and 9°C, and an average monthly temperature which varies in the year between 0.3°C and 11.8°C. It experiences high levels of precipitation throughout the year, with an average rainfall between 2000 and 4000 mm per year. Precipitation levels are higher in the winter than in the summer [35]. The area is coastal with a rocky substrate. The vegetation is abundant and is characterized by a forest type known as Valdivian rainforest and Norpatagonicus Valdivian rainforest. The arboreal stratum consists of perennial species such as *Notofagus dombeyi*, *Drymis winteri*, and *Laureliopsis philipiana*, among others. The understory is mainly composed of species such as *Luma apiculata*, *Quila chusquea*, and *Fuchsia magellanica* [36, 37]. At least 117 people are living in Puerto Gaviota which is 40 km from the study site (Seno Magdalena) [35]. There is also a salmon farming center in Seno Magdalena, inhabited by no more than 15 people. Area 4 is currently the only location where there are no domestic cats. The high heterogeneity of ecosystems along the four study areas is due to strong variations in latitude and altitude in addition to environmental disturbances such as volcanoes, glaciers, and landslides that contribute to biological diversity [36].

### Animals and samples

From 2009 to 2013, American mink (n = 73) were captured using modified Tomahawk traps with double doors [38]. Once captured, animals were immobilized mechanically with a plastic mesh and then chemically with an intramuscular (IM) administration of 10 mg/kg of Ketamine (Ketamina 100®, Chemie) and 0.025 mg/kg of Dexmedetomidine (Dexdomitor®, Pfizer). Blood samples were obtained though intracardiac puncture. After blood sampling, all mink were euthanized with thiopental (Tiopental Sódico®, Chemie). All animals were necropsied and tissues samples from the brain, lung, liver, and kidney were obtained. In total, tissues samples from 55 animals were collected. Otters (n = 13) were captured in leg hold soft-catch traps [39, 40]. After manual immobilization, a combination of Ketamine:dexmedetomidine 5:0.025 mg/kg IM was injected. Blood samples were obtained from a jugular vein. Once the individuals were microchipped and fully recovered from anesthesia they were released at the site of capture. Cats (n = 65) were sampled near the area where mink and otters were trapped. With the owner’s consent, each cat was manually immobilized to obtain a blood sample from a jugular vein. During the field work, two kodkod were accidentally captured and thus also sampled, one in Neltume and one in Puyuhuapi. In addition, a dead otter from Valdivia was also examined. All blood samples were obtained using sterile syringes and collected in 2 ml tubes with and without anticoagulant. To obtain serum, the blood without anticoagulant was centrifuged at 1200 g for 10 minutes. All samples were stored in liquid nitrogen until analysis. The tissue samples were preserved in alcohol 70% to preserve the tissues and ensure readability. Capture and sampling methods was carried out in strict accordance with the recommendations by Bioethics Committee for Animal Research of the Universidad Andres Bello and National Commission for Science and Technology. The field permit was granted by Subsecretaría de Pesca (Subpesca) for otters. Mink euthanasia was ordered by Servicio Agrícola y Ganadero (SAG). Sampling procedures in the field were reviewed and approved by veterinarians. All efforts were made to minimize suffering. Animal research ethics committee prospectively approved this research.

### Serological analysis

Sera from American mink, otter, domestic cat, and kodkod were evaluated for the detection of IgG antibodies against *T*. *gondii* using the modified agglutination test (MAT) [41]. MAT is one of the most evaluated and widely used tests both in clinical and epidemiological surveys for the detection of *T*. *gondii* in several animal species, including humans [5]. Sera were tested at 1:25, 1:50, 1:100, and 1:500 dilutions. Titers ≥25 were considered positive and those with doubtful results were re-tested. Positive and negative controls were included in this test. Additionally, we used Toxo-Screen DA fast kit [42] to as a complementary test of MAT in 40 samples of wild animals (29 American mink and 11 otter, were also included in MAT). Toxo-Screen DA fast kit, allows detection of IgG by direct agglutination. Sera were tested at 1:40 and 1:4000 dilutions. Titers ≥40 were considered positive [42]. The MAT and Toxo-Screen DA kit use same procedures and reagent [5].

### Molecular analysis

Tissue samples (lung, brain, liver, or kidney) from American mink and from one otter found dead in Valdivia were tested for the presence of *T*. *gondii* DNA. DNA was extracted using a commercial kit, NucleoSpin Tissue (Macherey-Nagel, Duren, Germany) according to the manufacturer's procedure. The extracted DNA was amplified using a real-time PCR [43] with Toxo-SE (5' AGGCGAGGGTGAGGATGA 3') and Toxo -AS (5' TCGTCTCGTCTGGATCGCAT 3') primers and the probe (5' 6FAM—CGACGAGAGTCGGAGAGGGAGAAGATGT—BHQ1 3') using a commercial kit (TaqMan PCR Master Mix, Applied Biosystems, Carlsbad, CA, USA).

### Domestic cat presence

Cat and human presence was determined via field trapping, human surveys, and spatial analysis using Quantum GIS version 2.0.1 (http://www.qgis.org/es/site/). To establish our study sites, we made a buffer area of 4 km around each American mink captured, individual buffers were then merged to produce a wider area, creating 11 study sites with a similar size. Buffered areas were divided in 1 km^2^ cells, which were then categorized as containing or not containing human presence. Google Earth (https://www.google.com/earth/) imagery was used to determine the presence of houses via visual identification of roofs, and cells with one or more houses were designated as having human presence and cells without houses were designated as not having human presence. Then, we counted the number of roofs per cell to have an approximate number of houses per site as a proxy of human presence or anthropogenic disturbance. Additionally, we conducted an on-ground survey for home owners to estimate the number of cats per house detected in Google Earth. Data of human presence and cats per house were used in posterior analyses as raw-continuous and categorical variables. Estimated number of cats per cell were divided in three categories to denote areas of low (≤0.2 cats per km^2^), medium (0.21–0.6 cats per km^2^), and high (0.61–5.5 cats per km^2^) degree of cat density.

### Land cover change

We explored the potential effects of anthropogenic land use change on the occurrence of *T*. *gondii* in the wild species used as sentinels. Land cover change was estimated via comparing the vegetation change in a ten-year period. MODIS imagery from the Terra satellite at 500 m. spatial resolution (MOD13A1.005) was used to obtain information of the landscape configuration. Specifically, employing 16-day composites Enhanced Vegetation Index (EVI) variables for February 2002 and 2012, the last representing the sampling period. The open access variables [44] were first processed using the MODIS Reprojection Tool version 4.1 to obtain raster files for posterior analyses [44]. Rasters were then analyzed using a principal components analysis in ArcGIS 10.3 [45]. The second component was used to identify areas of vegetation loss and gain. The second principal component from original vegetation indices is an excellent proxy of landscape variation [46]. This component was visually inspected to corroborate areas with loss and gain of vegetation.

### Statistical analysis

The differences in observed seroprevalence between species host (mink, otter and cat), study sites, then study areas and at the end habitat (Freshwater, Coastal, Island) as predictors, were assessed by Generalised Linear Models (GLM) using SYSTAT [47]. Separately, categorical variables of single differences between sex, age, cat, and human density (Low, Medium, High) within each species were assessed by Nonparametric Kruskal-Wallis test [48], as data fail to be independent. A *P* <0.05 value was considered statistically significant. To strengthen this assessment, a complementary analysis was conducted using all the variables to identify their importance to explain the presence of toxoplasmosis. We analyzed all variables including categorical (age, species, habitat type, sex) and the original continuous variables (cats density, human presence). This analysis was developed using a machine learning approach; specifically, we used a random forest algorithm due to its abilities to analyze continuous and categorical variables and flexibility to perform several types of statistical evaluations, including regressions, classification, and supervised learning [49]. The analysis was done using the caret package in R [50], following protocols described elsewhere [51]. To compare the two serological tests we used Cohen’s kappa analysis [52]. Finally, to assess the effect of Land Cover Change we used the locations of otter and mink captured, *T*. *gondii* positive and negative, to assess binary association of *T*. *gondii* in wildlife with continuous values landscape variation (i.e., principal component two of EVI data). This analysis was done separately from the previous ones using a logistic regression in R [53].

## Results

**Fig 2:**
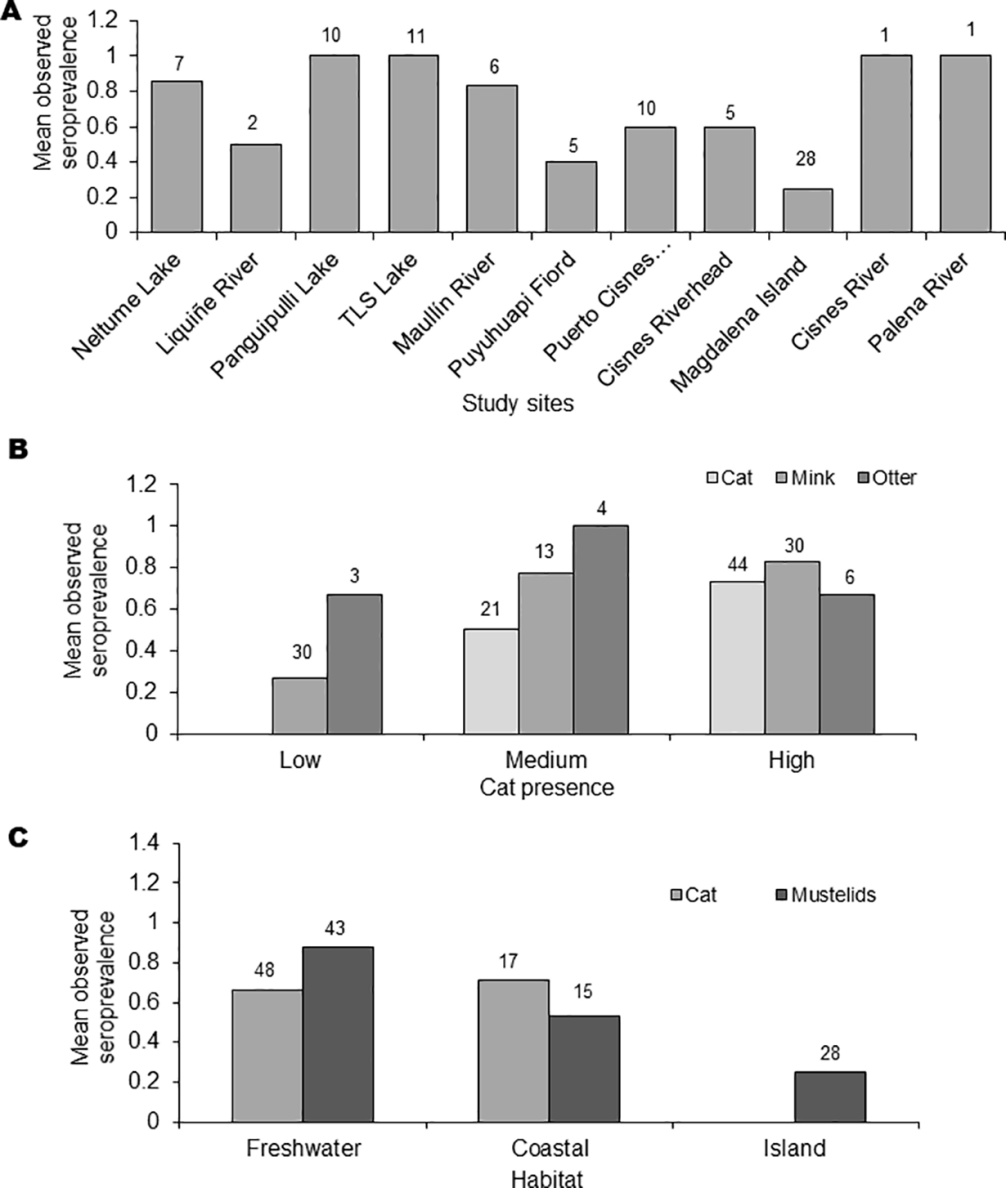
Effects of landscape variables on seroprevalence. A) Observed seroprevalence in mink and otter by study site. B) Observed seroprevalence by degree of presence of domestic cat. C) Observed seroprevalence by habitat. * Number above bars indicate sample size. Y axis show mean seroprevalence.

**Fig 3:**
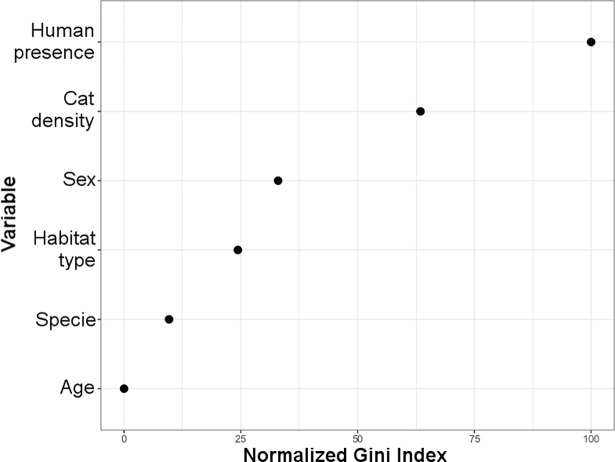
Variable importance analysis performed using random forest. The set of four categorical variables (age, species, habitat type, sex) and two continuous variables (human presence and cat density) used for classification the seropositivity to *Toxoplasma gondii*. Variables are ordered by their importance from top to bottom as estimated by the random forest model and denoted using an Gini index ranging from 0 to 100.

**Fig 4:**
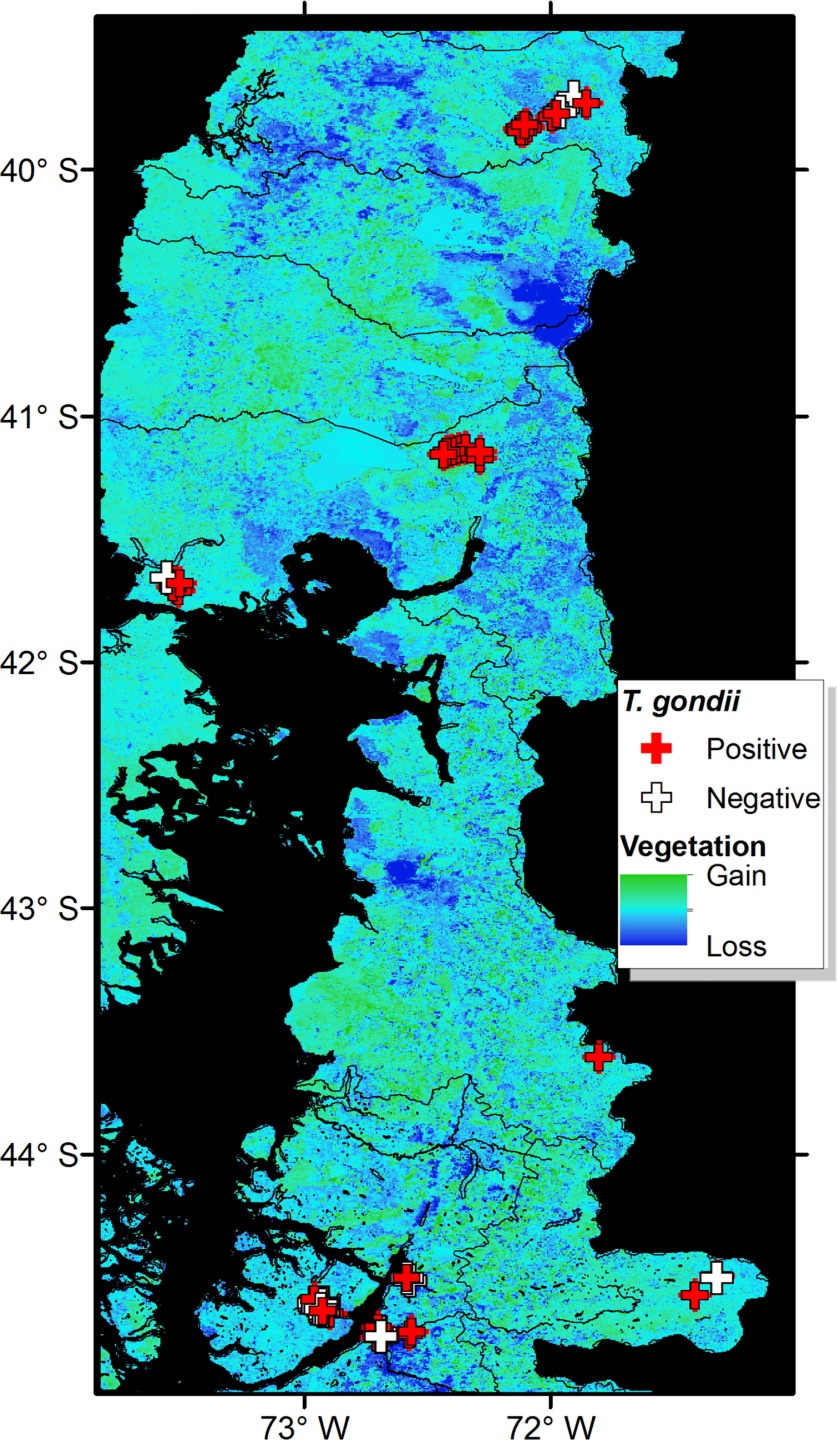
Land cover change and *Toxoplasma gondii* reports. Positive (red crosses) and negative (white crosses) reports of *T*. *gondii* in wildlife compared with vegetation change represented in the second principal component of the EVI 2002 and 2012. Change was denoted as areas of vegetation gain (green) and stability (light blue), and considerable vegetation loss (dark blue).

The overall *T*. *gondii* seroprevalence, according to the MAT analysis, was 64% (97/151), with 68% (44/65) in domestic cats, 59% (43/73) American mink, and 77% (10/13) in otter (Table 1). The two kodkods were also seropositive. With GLM and Kruskal-Wallis test, seroprevalence for *T*. *gondii* among study sites showed no statistical differences (Fig 2A). However, there were differences between study areas (F_3,147_ = 9.7; P<0.01) because the significantly lower prevalence in area 4 (Magdalena Island), there were not significantly difference between the other areas (F_2,120_ = 1.12; P = 0.33). Analysis by species showed a consistent prevalence in cats among all of the analyzed study sites, areas and habitats, but not in Magdalena island. No differences were found between *T*. *gondii* seroprevalences of mink and otter, or between animal sex or age, although a tendency towards higher prevalence was observed in older animals. Mink had significantly higher seroprevalences in the study sites associated to medium and high presence of domestic cats (H_2_ = 22.0; P<0.01) (Fig 2B), and significantly less observed seroprevalences in marine habitats (H_2_ = 28.9; P<0.01) (Fig 2C). In fact, the only study site with significantly less observed seroprevalence was Magdalena Island (Fig 2A). Otter seropositivity had no relationship with cat presence, but was also significantly lower in marine habitats (H_1_ = 31.5; P = 0.04) (Fig 2C). Indeed, the differences of seroprevalences between study sites for otters were close to significant (F_5,7_ = 3.5; P = 0.07). Our random forest analysis revealed that seropositivity to *Toxoplasma gondii* was mainly explained by presence of humans and density of cats (Fig 3). *Toxoplasma gondii* DNA was detected in the liver of one American mink in *Choshuenco* (Area 1), and in the lung of the otter found dead in Valdivia (outside of the study areas). The analysis of the vegetation structure revealed considerable variation in the vegetation biomass in the ten-year period assessed (Fig 4). At province level, areas of dramatic vegetation loss were identified in central western Valdivia, eastern Ranco, dispersed areas across Llanquihue, central west Palena, and across the southern sampled areas in Aysen. A significant association was found between the *T*. *gondii* seropositivity and the level of vegetation loss of the study sites (COR = 0.65; df = 85; *P*<0.001) with habitat loss being associated with increase of *T*. *gondii* seroprevalence. Results obtained by Toxo-Screen DA fast kit were similar to those obtained with MAT. Of the 40 samples, 27 were equally positive and 9 equally negative, with 2 “false positives” and 2 “false negatives”. The Cohen’s kappa analysis shows a good agreement between the two tests (Kappa = 0.75; 95% confidence interval = 0.52–0.98; *P*<0.001).

**Table 1:** Seroprevalence of *Toxoplasma gondii*. Data from 11 study sites with varying degrees of domestic cat presence, (NS = Not samples).

Study Region	Sites	Cat density	Prevalence % (positive/assayed)			
			Cat	Mink	Otter	Total
Area 1—Andean foothill			65.71 (23/35)	91.30 (21/23)	100 (7/7)	78.46 (51/65)
Liquiñe		High	80 (8/10)	50 (1/2)	NS	75 (9/12)
Neltume		High	44.44 (4/9)	83.33 (5/6)	100 (1/1)	62.5 (10/16)
Choschuenco		High	81.81 (9/11)	100 (7/7)	100 (3/3)	90.48 (19/21)
Todos los Santos Lake		Medium	40 (2/5)	100 (8/8)	100 (3/3)	81.25 (13/16)
Area 2 –Marine coastal ecosystem			67.86 (19/28)	68.42 (13/19)	33.33 (1/3)	65.45 (33/50)
Puerto Cisnes		High	50 (14/18)	75 (6/8)	0 (0/2)	71.43 (20/28)
Maullín		High	0 (0/1)	83.33 (5/6)	NS	71.43 (5/7)
Puyuhuapi		Medium	50 (3/6)	25 (1/4)	100 (1/1)	45.45 (5/11)
Rio Cisnes		Medium	66.66 (2/3)	100 (1/1)	NS	75 (3/4)
Area 3 –Andean Mountain Valley			100 (2/2)	66.66 (4/6)		75 (6/8)
Palena		High	100 (2/2)	100 (1/1)	NS	100 (3/3)
Alto Rio Cisnes		Low	NS	60 (3/5)	NS	60 (3/5)
Area 4 –Island coastal						
Isla Magdalena		Low	NS	20 (5/25)	66.66 (2/3)	25 (7/28)
TOTAL			67.69 (44/65)	58.90 (43/73)	76.92 (10/13)	64.24 (97/151)

## Discussion

The present study confirms the widespread distribution of *T*. *gondii* in Southern Chile, and shows a high exposure of semiaquatic mustelids and domestic cats to the parasite. The high explanatory power of human presence followed of cat density in our machine learning analysis (Fig 3), suggests the role of domestic cats as a form of anthropogenic disturbance for the maintenance of *T*. *gondii* infections in Southern Chilean ecosystems. The mean prevalences observed in the study, 59% in mink, 77% in otters, and 68% in domestic cats are consistent with those observed in previous studies. A 70% prevalence of *T*. *gondii* in American mink has been reported in the Maullín River [32]. We observed similarly high seroprevalence level at the same site in mink in the current study (83%). Our findings also agree with recent reports of *T*. *gondii* seroprevalences of 77% in American mink in the USA [54]. Seroprevalence of *T*. *gondii* revealed the lowest values (25%) in Magdalena Island. These results were expected considering that there are no reports of the presence of domestic cats in this island due to its geographic isolation (five km distant from the continent). Our results also agree with another study in USA which suggests that prevalence of *T*. *gondii* antibodies below 40% indicates low exposure to the parasite [55]. However, the prevalence on this island may support the idea of horizontal transmission of *T*. *gondii*. Mink and otters from Magdalena Island may have become infected while feeding on other intermediate hosts.

Studies of the mechanisms of *T*. *gondii* transmission in islands are of great interest since isolated environments can affect the epidemiology of different pathogens. In this respect, several studies have reported positive relationship between prevalences of *T*. *gondii* in intermediate hosts and the level of the presence of cats. However, the presence of the parasite in islands without felids has been reported in Arctic foxes (*Vulpes lagopus*) from Svalbard Island [42]. *Toxoplasma gondii* seroposivity of seagull chicks in an island without cats has also been reported, confirming the capability of seagulls to introduce pathogens in naïve territories and expose other susceptible hosts [16]. Sites with moderate *T*. *gondii* prevalence (i.e., 40–60%) [55] were Puyuhuapi Fiord (45.4%) which is about 20 km from the nearest village, and Alto-Rio-Cisnes (60%). All other sites showed high seroprevalences (>60%; Fig 2A).

As reported previously, we observed lower seroprevalence in wildlife from study sites with low degree of housing and therefore low human presence. However, this result was not observed in cats. Domestic cats seroprevalence did not show differences based on cat density (Fig 2B, Table 1). This may be explained by the role of the felids in the epidemiological cycle of *T*. *gondii*. The domestic cat is the key species for the maintenance of *T*. *gondii* oocyts in the environment, excreting millions of oocysts in a short period of time [56]. It has been estimated that between the 1% and 2% of all domestic cats excrete *T*. *gondii* oocysts at any given time [57, 58, 59].

In Chile, feral cat populations are scattered and found mainly in Central-South regions; thus, cats have sympatric distribution with the otter and the American mink [60]. In rural areas of Central Chile, up to 54.6% seropositivity against *T*. *gondii* has been recorded in domestic cats [61] while in the Southern areas, i.e. in the city of Valdivia, 33% [62]. Recently, in a smaller city, the city of San Carlos, north of Valdivia, a seroprevalence of 48.3% was reported in domestic cats, suggesting a consistent range of *T*. *gondii* prevalence in the area [63]. Thus, the numbers of oocysts released to the environment is not dependent on the cat population density (cats per area), but on their abundance (total number of cats). Therefore, we did not expect to find significant differences in prevalence among domestic cat populations along a certain territory. Contrary, if there is an important participation of the cat’s population in a process of pathogen pollution, we expect higher levels of *T*. *gondii* in the environment where there are larger populations of cats [64].

As an example of environmental contamination, in North Carolina, USA, higher seroprevalences of *T*. *gondii* were found in feral cats than in pet cats, and higher seroprevalences in pet cats that had access to outdoors than those that did not [65]. Nevertheless research on *T*. *gondii* in native wild felids has been limited even considering their crucial role in the ecology and epidemiology of toxoplasmosis [66, 67]. In southern Brazil, *T*. *gondii* seroprevalences ranged between 14% to 100% in six free ranging wild small neotropical feline species [68]. The present study only tested two kodkods and both were seropositive.

We recorded higher seroprevalence in native and invasive wild species (semiaquatic carnivores) in three areas than that of domestic cats (Fig 2B). Higher levels of infection of *T*. *gondii* in semiaquatic than terrestrial mammals have been reported recently elsewhere [54]. These results support the hypothesis that American mink and otter may be consuming oocysts directly from contaminated water or filter feeders such as mussels as previously reported [69, 70]. The spatial patterns of prevalence in the American mink offer a proxy for the oocyst load in coastal waters [55]. Nevertheless, mink also predate on mice and rats [71, 72]. Thus, mink may be competing with domestic cats and wild cats for prey. Furthermore, the association found between the occurrence of *T*. *gondii* and landscape change suggests that habitat loss may also affect the ecology of *T*. *gondii*, for example, altering small mammal communities [73, 74] and also altering runoff and flooding. Under this scenario, the effects of native forest loss, increase farming, together with the invasion of mink on small mammal communities should be studied in Patagonia.

*Toxoplasma gondii* DNA was detected in liver tissue from an American mink and in lung tissue from an otter. Interestingly, the presence of the parasite in these tissues may indicate an acute disseminated disease in these animals. Protozoan parasites such as *T*. *gondii* have been proven to compromise the viability of some aquatic mustelid populations in other geographic areas [18, 19]. In this respect, it is important to evaluate the effect of *T*. *gondii* exposure of wild endangered species populations from Chile.

Our study shows novel data of the distribution of *T*. *gondii* among native and non-native carnivore species in ecosystems of southern Chile with different levels of anthropogenic perturbation and domestic cat abundance. We argue that by reducing the abundances of domestic cats, and restoring the areas of vegetation loss would improve the status of the native biodiversity, but most importantly would result in a reduction of risk of exposure of humans to *T*. *gondii*. In conclusion, toxoplasmosis is a model disease to be included in the One Health diary as it may affect the environmental, animal, and human health. And also as our results suggest Toxo-Screen DA could be a good diagnostic tool considering how fast and easy is to implement.

## Supporting information

S1 Protocol (PDF)

S1 File (XLSX)

S2 File (7Z)
