# Supplementary material for: Toxoplasma gondii infection in wild mustelids and cats across an urban-rural gradient
Source: PLoS One. 2018 Jun 20;13(6):e0199085. doi: 10.1371/journal.pone.0199085 (PMC6010287; doi:10.1371/journal.pone.0199085)
Supplement: S1 Protocol — (PDF) [file pone.0199085.s001.pdf]

TOXOPLASMA AGGLUTINATION TEST

1. SERUM DILUTING BUFFER
Dissolve 42.5 g NaCl, 1.54 g NaH_2PO_4, and 5.4 g Na_2HPO_4 in 900 ml deionized water. Adjust the pH to 7.2. Bring the volume to 1 liter with deionized water. Stored in a refrigerator. This is the 5X stock solution. Dilute this stock solution 1:5 to give 0.01 M phosphate buffered saline (PBS) (1 part stock and 4 parts deionized water). PBS should be filtered just before use through a 0.22 μM membrane.

2. ANTIGEN DILUTING BUFFER
Dissolve 7.01 g sodium chloride, 3.09 g boric acid, 2.0 g sodium azide in 900 ml deionized water. Add 24 ml 1 N NaOH and adjust the pH to 8.95. Bring the volume to 1 liter. This is the stock solution and can be stored at room temperature. For the working antigen diluting buffer, dissolve 0.4 g bovine serum albumin (BSA) in 100 ml borate buffer. Stored refrigerated.
(stock solution)

3. Dilute serum samples with serum diluting buffer (1) in small test tubes (1.2 ml in strips of 8 or 12) with a multichannel pipette, starting at 1:25. Microtiter plates may also be used for making serum dilutions.

4. Prepare antigen mixture as follows:
For each plate, mix 2.5 ml antigen diluting buffer (2), 35 μl 2-mercaptoethanol, 50 μl Evans blue dye solution (2 mg/ml water) and 0.15 ml antigen (formalin-fixed whole parasites).
(150 µl)

5. Agglutination is done in U bottom 96 well microtiter plates. Pipet 25 μl antigen mixture to each well immediately after mixing. Pipet 25 μl serum dilutions into the wells and mix gently with the antigen by repeated pipetting action.

6. A positive control should be included in each plate. The control should have a titer of 1:200, and two-fold dilutions from 1:25 to 1:3200 should be used.

8. Cover the plates with sealing tape and incubate at 37° over night.

9. Read results. A blue button at the bottom of the well means negative. A clear bottom means positive.

sueros 10 µl / 240 SDB

SDB ≥ 20 ml / placa

TOXOTEST.DOC
revised 98.01.26

Solo 20 µl / 480 SDB para filtrar
